# 2,4-Diiodo-6-{[4-(morpholin-4-yl)phenyl]iminomethyl}phenol

**DOI:** 10.1107/S1600536811034519

**Published:** 2011-08-27

**Authors:** K. Manvizhi, G. Chakkaravarthi, G. Anbalagan, G. Rajagopal

**Affiliations:** a Department of Chemistry, Anand Institute of Higher Technology, Kazhipattur, Chennai 603 103, India; b Department of Physics, CPCL Polytechnic College, Chennai 600 068, India; c Department of Physics, Presidency College (Autonomous), Chennai 600 005, India; d Department of Chemistry, Government Arts College, Melur 625 106, India

## Abstract

In the title compound, C_17_H_16_I_2_N_2_O_2_, the two aromatic rings are almost coplanar [dihedral angle 2.57 (15)°]. The morpholine ring adopts a chair conformation. The mol­ecular structure is stabilized by an O—H⋯N hydrogen bond and the crystal packing exhibits weak inter­molecular C—H⋯O and π–π [centroid-to-centroid distances 3.663 (3)-4.073 (3) Å] inter­actions.

## Related literature

For the biological activity of morpholine derivatives, see: Lan *et al.* (2010 ▶); Raparti *et al.*(2009 ▶). For a related structure, see: Yang *et al.* (2011 ▶). For the definition of puckering parameters, see: Cremer & Pople (1975 ▶).
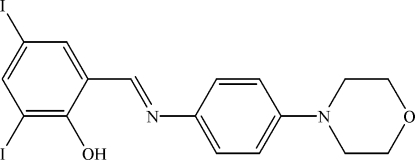


## Experimental

### 

#### Crystal data

C_17_H_16_I_2_N_2_O_2_
*M* *_r_* = 534.12
Monoclinic, 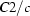

*a* = 26.4133 (16) Å
*b* = 7.6598 (4) Å
*c* = 18.0332 (11) Å
β = 91.417 (2)°
*V* = 3647.4 (4) Å^3^
*Z* = 8
Mo *K*α radiation
μ = 3.46 mm^−1^
*T* = 295 K
0.26 × 0.20 × 0.20 mm

#### Data collection

Bruker Kappa APEXII diffractometer
Absorption correction: multi-scan (*SADABS*; Sheldrick, 1996 ▶) *T*_min_ = 0.467, *T*_max_ = 0.545
17839 measured reflections
7647 independent reflections
4855 reflections with *I* > 2σ(*I*)
*R*_int_ = 0.023

#### Refinement

*R*[*F* ^2^ > 2σ(*F* ^2^)] = 0.056
*wR*(*F* ^2^) = 0.113
*S* = 1.16
7647 reflections
208 parameters
H-atom parameters constrained
Δρ_max_ = 1.22 e Å^−3^
Δρ_min_ = −1.72 e Å^−3^

### 

Data collection: *APEX2* (Bruker, 2004 ▶); cell refinement: *SAINT* (Bruker, 2004 ▶); data reduction: *SAINT*; program(s) used to solve structure: *SHELXS97* (Sheldrick, 2008 ▶); program(s) used to refine structure: *SHELXL97* (Sheldrick, 2008 ▶); molecular graphics: *PLATON* (Spek, 2009 ▶); software used to prepare material for publication: *SHELXL97*.

## Supplementary Material

Crystal structure: contains datablock(s) global, I. DOI: 10.1107/S1600536811034519/bt5621sup1.cif

Structure factors: contains datablock(s) I. DOI: 10.1107/S1600536811034519/bt5621Isup2.hkl

Supplementary material file. DOI: 10.1107/S1600536811034519/bt5621Isup3.cml

Additional supplementary materials:  crystallographic information; 3D view; checkCIF report

## Figures and Tables

**Table 1:** Hydrogen-bond geometry (Å, °)

*D*—H⋯*A*	*D*—H	H⋯*A*	*D*⋯*A*	*D*—H⋯*A*
O2—H2⋯N1	0.82	1.82	2.548 (5)	146
C6—H6⋯O1^i^	0.93	2.50	3.413 (5)	166

Symmetry code: (i) 
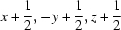
.

**Fig. 1.**
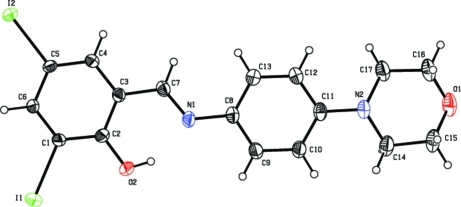
The molecular structure of the title compound, with atom labels and 30% probability displacement ellipsoids for non-H atoms.

**Fig. 2.**
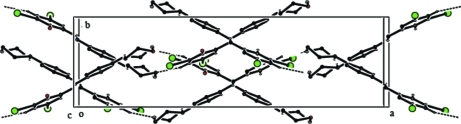
The packing of the title compound, viewed down the c axis. Intermolecular hydrogen bonds are shown as dashed lines. H atoms not involved in hydrogen bonding have been omitted.

## supplementary crystallographic information

### Comment

Morpholine derivatives possess anticancer and antimicrobial (Lan *et al.*, 2010; Raparti *et al.*, 2009) activities. In the title compound, (I) (Fig. 1), The bond lengths C═N [1.282 (6)Å], C—I [C1—I1 = 2.092 (4) and C5—I2 = 2.097 (4) Å] are comparable with the literature values and the bond lengths of the morpholine ring are agree well with a reported related structure (Yang *et al.*, 2011).

The mean planes of two benzene rings (C8–C13) and (C1–C6) are oriented at an angle of 2.57 (15)°. The morpholine ring adopts a chair conformation [Puckering parameters are Q = 0.544 (6)Å, θ = 170.8 (5)° and φ = 180 (4)° (Cremer & Pople, 1975) for the ring (O1/C15/C14/N2/C17/C16)].

The molecular structure is stabilized by O—H···N hydrogen bonding and the crystal packing exhibit weak intermolecular C—H···O (Fig. 2 and Table 1) and π–π [Cg2···Cg3(-x, -y, -z) distance of 3.663 (3)Å; Cg2···Cg3(-x, 1 - y, -z) distance of 4.074 (3)Å] interactions.

### Experimental

An ethanolic solution (20 ml) of 4-(4-aminophenyl)morpholine (10 mmol) was magnetically stirred in a round bottom flask followed by drop wise addition of ethanolic solution of 3,5-diiodosalicylaldehyde (10 mmol). The reaction mixture was then refluxed for 3 h and upon cooling to 273 K, a red crystalline solid precipitates from the mixture. The solid which is separated out was filtered washed with ice cold ethanol and dried in vaccuo over anhydrous CaCl_2_. Single crystals suitable for the X-ray diffraction were obtained by slow evaporation of a solution of the title compound in methanol at room temperature. m.p. 443 K.

### Refinement

All H atoms were positioned geometrically with C—H = 0.93–0.97 Å and O—H = 0.82 Å and allowed to ride on their parent atoms, with U_iso_(H) = 1.5Ueq(O) and 1.2Ueq(C).

### Crystal data

**Table:**

C_17_H_16_I_2_N_2_O_2_	*F*(000) = 2032
*M**_r_* = 534.12	*D*_x_ = 1.945 Mg m^−^^3^
Monoclinic, *C*2/*c*	Mo *K*α radiation, λ = 0.71073 Å
Hall symbol: -C 2yc	Cell parameters from 6743 reflections
*a* = 26.4133 (16) Å	θ = 2.7–35.4°
*b* = 7.6598 (4) Å	µ = 3.46 mm^−^^1^
*c* = 18.0332 (11) Å	*T* = 295 K
β = 91.417 (2)°	Block, colourless
*V* = 3647.4 (4) Å^3^	0.26 × 0.20 × 0.20 mm
*Z* = 8	

### Data collection

**Table:**

Bruker Kappa APEXII diffractometer	7647 independent reflections
Radiation source: fine-focus sealed tube	4855 reflections with *I* > 2σ(*I*)
graphite	*R*_int_ = 0.023
ω and φ scans	θ_max_ = 35.9°, θ_min_ = 2.3°
Absorption correction: multi-scan (*SADABS*; Sheldrick, 1996)	*h* = −42→42
*T*_min_ = 0.467, *T*_max_ = 0.545	*k* = −5→12
17839 measured reflections	*l* = −29→27

### Refinement

**Table:**

Refinement on *F*^2^	Primary atom site location: structure-invariant direct methods
Least-squares matrix: full	Secondary atom site location: difference Fourier map
*R*[*F*^2^ > 2σ(*F*^2^)] = 0.056	Hydrogen site location: inferred from neighbouring sites
*wR*(*F*^2^) = 0.113	H-atom parameters constrained
*S* = 1.16	*w* = 1/[σ^2^(*F*_o_^2^) + (0.0134*P*)^2^ + 27.6528*P*] where *P* = (*F*_o_^2^ + 2*F*_c_^2^)/3
7647 reflections	(Δ/σ)_max_ < 0.001
208 parameters	Δρ_max_ = 1.22 e Å^−^^3^
0 restraints	Δρ_min_ = −1.72 e Å^−^^3^

### Fractional atomic coordinates and isotropic or equivalent isotropic displacement parameters (Å^2^)

**Table:**

	*x*	*y*	*z*	*U*_iso_*/*U*_eq_	
I1	0.198728 (14)	−0.04313 (7)	0.01153 (2)	0.06368 (14)	
I2	0.097444 (12)	−0.01689 (5)	0.305807 (16)	0.04538 (10)	
O1	−0.25208 (14)	0.6183 (6)	−0.2595 (3)	0.0645 (11)	
O2	0.09297 (14)	0.1239 (6)	−0.03377 (17)	0.0567 (10)	
H2	0.0683	0.1836	−0.0453	0.085*	
N1	0.00453 (14)	0.2516 (5)	−0.0214 (2)	0.0399 (9)	
N2	−0.16015 (14)	0.5632 (5)	−0.1763 (2)	0.0425 (9)	
C1	0.13595 (16)	0.0208 (6)	0.0746 (2)	0.0369 (9)	
C2	0.09350 (17)	0.0948 (6)	0.0387 (2)	0.0373 (9)	
C3	0.05152 (15)	0.1370 (6)	0.0821 (2)	0.0335 (9)	
C4	0.05340 (16)	0.1049 (6)	0.1581 (2)	0.0361 (9)	
H4	0.0257	0.1332	0.1867	0.043*	
C5	0.09549 (15)	0.0320 (6)	0.1913 (2)	0.0329 (9)	
C6	0.13743 (15)	−0.0108 (6)	0.1495 (2)	0.0337 (9)	
H6	0.1660	−0.0601	0.1721	0.040*	
C7	0.00713 (17)	0.2167 (6)	0.0481 (3)	0.0395 (10)	
H7	−0.0203	0.2434	0.0775	0.047*	
C8	−0.03754 (17)	0.3331 (6)	−0.0573 (3)	0.0396 (10)	
C9	−0.03566 (18)	0.3557 (7)	−0.1329 (3)	0.0450 (11)	
H9	−0.0070	0.3202	−0.1577	0.054*	
C10	−0.07575 (19)	0.4305 (7)	−0.1727 (3)	0.0462 (11)	
H10	−0.0735	0.4440	−0.2238	0.055*	
C11	−0.11929 (17)	0.4858 (6)	−0.1375 (3)	0.0402 (10)	
C12	−0.12009 (19)	0.4645 (8)	−0.0609 (3)	0.0505 (13)	
H12	−0.1483	0.5023	−0.0355	0.061*	
C13	−0.0805 (2)	0.3894 (8)	−0.0215 (3)	0.0520 (13)	
H13	−0.0825	0.3762	0.0296	0.062*	
C14	−0.1627 (2)	0.5380 (8)	−0.2559 (3)	0.0591 (15)	
H14A	−0.1692	0.4159	−0.2668	0.071*	
H14B	−0.1305	0.5691	−0.2768	0.071*	
C15	−0.2046 (2)	0.6497 (9)	−0.2912 (4)	0.0682 (18)	
H15A	−0.1959	0.7720	−0.2852	0.082*	
H15B	−0.2069	0.6250	−0.3440	0.082*	
C16	−0.2480 (2)	0.6635 (9)	−0.1835 (4)	0.0655 (17)	
H16A	−0.2807	0.6489	−0.1611	0.079*	
H16B	−0.2385	0.7855	−0.1791	0.079*	
C17	−0.20944 (19)	0.5535 (8)	−0.1423 (3)	0.0573 (15)	
H17A	−0.2064	0.5932	−0.0913	0.069*	
H17B	−0.2208	0.4331	−0.1419	0.069*	

### Atomic displacement parameters (Å^2^)

**Table:**

	*U*^11^	*U*^22^	*U*^33^	*U*^12^	*U*^13^	*U*^23^
I1	0.04506 (19)	0.1026 (4)	0.04403 (18)	0.0186 (2)	0.01452 (14)	−0.0004 (2)
I2	0.04006 (15)	0.0669 (2)	0.02930 (13)	0.00174 (15)	0.00371 (10)	0.00684 (14)
O1	0.0412 (19)	0.067 (3)	0.084 (3)	−0.0058 (19)	−0.0255 (19)	0.011 (2)
O2	0.059 (2)	0.086 (3)	0.0255 (15)	0.027 (2)	0.0000 (14)	−0.0027 (17)
N1	0.0382 (19)	0.044 (2)	0.0372 (19)	0.0059 (17)	−0.0102 (16)	−0.0042 (17)
N2	0.0365 (19)	0.037 (2)	0.053 (2)	−0.0016 (16)	−0.0128 (17)	0.0076 (18)
C1	0.0314 (19)	0.050 (3)	0.0293 (18)	0.0038 (19)	0.0014 (15)	−0.0053 (19)
C2	0.039 (2)	0.045 (3)	0.0277 (19)	0.000 (2)	−0.0013 (16)	−0.0041 (18)
C3	0.0301 (19)	0.038 (2)	0.0325 (19)	0.0010 (17)	−0.0032 (16)	−0.0035 (17)
C4	0.0287 (19)	0.044 (3)	0.035 (2)	0.0003 (18)	0.0036 (16)	0.0006 (19)
C5	0.0327 (19)	0.041 (2)	0.0249 (16)	−0.0051 (18)	0.0025 (14)	0.0026 (17)
C6	0.0268 (17)	0.043 (2)	0.0316 (18)	0.0002 (17)	−0.0023 (14)	0.0017 (18)
C7	0.031 (2)	0.043 (3)	0.044 (2)	0.0021 (19)	−0.0029 (18)	−0.004 (2)
C8	0.035 (2)	0.039 (2)	0.044 (2)	0.0025 (19)	−0.0085 (18)	−0.001 (2)
C9	0.039 (2)	0.051 (3)	0.046 (3)	0.008 (2)	−0.004 (2)	−0.002 (2)
C10	0.044 (3)	0.052 (3)	0.042 (2)	0.007 (2)	−0.008 (2)	0.000 (2)
C11	0.034 (2)	0.034 (2)	0.052 (3)	−0.0049 (18)	−0.0112 (19)	0.004 (2)
C12	0.037 (2)	0.062 (3)	0.052 (3)	0.010 (2)	−0.002 (2)	0.004 (3)
C13	0.046 (3)	0.069 (4)	0.042 (3)	0.010 (3)	−0.002 (2)	0.006 (3)
C14	0.049 (3)	0.067 (4)	0.060 (3)	0.000 (3)	−0.016 (3)	0.014 (3)
C15	0.056 (3)	0.075 (4)	0.072 (4)	0.003 (3)	−0.024 (3)	0.019 (3)
C16	0.039 (3)	0.071 (4)	0.085 (5)	0.005 (3)	−0.017 (3)	0.012 (4)
C17	0.037 (2)	0.062 (4)	0.073 (4)	0.003 (2)	−0.010 (2)	0.011 (3)

### Geometric parameters (Å, °)

**Table:**

I1—C1	2.092 (4)	C8—C9	1.376 (7)
I2—C5	2.097 (4)	C8—C13	1.387 (7)
O1—C15	1.412 (7)	C9—C10	1.389 (6)
O1—C16	1.415 (8)	C9—H9	0.9300
O2—C2	1.325 (5)	C10—C11	1.393 (7)
O2—H2	0.8200	C10—H10	0.9300
N1—C7	1.282 (6)	C11—C12	1.392 (7)
N1—C8	1.417 (6)	C12—C13	1.376 (7)
N2—C11	1.403 (6)	C12—H12	0.9300
N2—C14	1.447 (7)	C13—H13	0.9300
N2—C17	1.455 (7)	C14—C15	1.525 (7)
C1—C6	1.372 (6)	C14—H14A	0.9700
C1—C2	1.401 (6)	C14—H14B	0.9700
C2—C3	1.411 (6)	C15—H15A	0.9700
C3—C4	1.393 (6)	C15—H15B	0.9700
C3—C7	1.445 (6)	C16—C17	1.504 (7)
C4—C5	1.369 (6)	C16—H16A	0.9700
C4—H4	0.9300	C16—H16B	0.9700
C5—C6	1.394 (6)	C17—H17A	0.9700
C6—H6	0.9300	C17—H17B	0.9700
C7—H7	0.9300		
			
C15—O1—C16	107.7 (4)	C11—C10—H10	119.4
C2—O2—H2	109.5	C12—C11—C10	116.7 (4)
C7—N1—C8	124.1 (4)	C12—C11—N2	120.9 (5)
C11—N2—C14	117.0 (4)	C10—C11—N2	122.3 (5)
C11—N2—C17	117.0 (4)	C13—C12—C11	122.1 (5)
C14—N2—C17	113.0 (4)	C13—C12—H12	118.9
C6—C1—C2	122.0 (4)	C11—C12—H12	118.9
C6—C1—I1	119.4 (3)	C12—C13—C8	120.7 (5)
C2—C1—I1	118.6 (3)	C12—C13—H13	119.7
O2—C2—C1	120.9 (4)	C8—C13—H13	119.7
O2—C2—C3	121.3 (4)	N2—C14—C15	110.8 (5)
C1—C2—C3	117.8 (4)	N2—C14—H14A	109.5
C4—C3—C2	119.8 (4)	C15—C14—H14A	109.5
C4—C3—C7	120.1 (4)	N2—C14—H14B	109.5
C2—C3—C7	120.2 (4)	C15—C14—H14B	109.5
C5—C4—C3	120.8 (4)	H14A—C14—H14B	108.1
C5—C4—H4	119.6	O1—C15—C14	112.2 (5)
C3—C4—H4	119.6	O1—C15—H15A	109.2
C4—C5—C6	120.5 (4)	C14—C15—H15A	109.2
C4—C5—I2	120.2 (3)	O1—C15—H15B	109.2
C6—C5—I2	119.3 (3)	C14—C15—H15B	109.2
C1—C6—C5	119.2 (4)	H15A—C15—H15B	107.9
C1—C6—H6	120.4	O1—C16—C17	112.1 (5)
C5—C6—H6	120.4	O1—C16—H16A	109.2
N1—C7—C3	121.7 (4)	C17—C16—H16A	109.2
N1—C7—H7	119.1	O1—C16—H16B	109.2
C3—C7—H7	119.1	C17—C16—H16B	109.2
C9—C8—C13	118.1 (4)	H16A—C16—H16B	107.9
C9—C8—N1	117.4 (4)	N2—C17—C16	111.4 (5)
C13—C8—N1	124.4 (4)	N2—C17—H17A	109.3
C8—C9—C10	121.3 (5)	C16—C17—H17A	109.3
C8—C9—H9	119.4	N2—C17—H17B	109.3
C10—C9—H9	119.4	C16—C17—H17B	109.3
C9—C10—C11	121.1 (5)	H17A—C17—H17B	108.0
C9—C10—H10	119.4		
			
C6—C1—C2—O2	−179.9 (5)	N1—C8—C9—C10	178.4 (5)
I1—C1—C2—O2	0.0 (7)	C8—C9—C10—C11	0.1 (8)
C6—C1—C2—C3	0.1 (7)	C9—C10—C11—C12	0.9 (8)
I1—C1—C2—C3	180.0 (3)	C9—C10—C11—N2	179.4 (5)
O2—C2—C3—C4	−179.9 (5)	C14—N2—C11—C12	−163.6 (5)
C1—C2—C3—C4	0.1 (7)	C17—N2—C11—C12	−24.8 (7)
O2—C2—C3—C7	−1.2 (7)	C14—N2—C11—C10	17.9 (7)
C1—C2—C3—C7	178.8 (4)	C17—N2—C11—C10	156.7 (5)
C2—C3—C4—C5	−0.2 (7)	C10—C11—C12—C13	−1.3 (8)
C7—C3—C4—C5	−178.9 (4)	N2—C11—C12—C13	−179.9 (5)
C3—C4—C5—C6	0.2 (7)	C11—C12—C13—C8	0.7 (9)
C3—C4—C5—I2	−179.6 (3)	C9—C8—C13—C12	0.4 (8)
C2—C1—C6—C5	−0.1 (7)	N1—C8—C13—C12	−178.8 (5)
I1—C1—C6—C5	180.0 (3)	C11—N2—C14—C15	−172.1 (5)
C4—C5—C6—C1	−0.1 (7)	C17—N2—C14—C15	47.5 (6)
I2—C5—C6—C1	179.8 (3)	C16—O1—C15—C14	61.6 (7)
C8—N1—C7—C3	−178.5 (4)	N2—C14—C15—O1	−55.3 (7)
C4—C3—C7—N1	178.5 (5)	C15—O1—C16—C17	−61.9 (6)
C2—C3—C7—N1	−0.2 (7)	C11—N2—C17—C16	171.5 (5)
C7—N1—C8—C9	−177.4 (5)	C14—N2—C17—C16	−48.1 (7)
C7—N1—C8—C13	1.7 (8)	O1—C16—C17—N2	55.7 (7)
C13—C8—C9—C10	−0.8 (8)		

### Hydrogen-bond geometry (Å, °)

**Table:**

*D*—H···*A*	*D*—H	H···*A*	*D*···*A*	*D*—H···*A*
O2—H2···N1	0.82	1.82	2.548 (5)	146
C6—H6···O1^i^	0.93	2.50	3.413 (5)	166

Symmetry codes: (i) *x*+1/2, −*y*+1/2, *z*+1/2.
